# Drug-induced Parkinson’s disease modulates protein kinase A and Olfactory Marker Protein in the mouse olfactory bulb

**DOI:** 10.1186/s12993-017-0119-2

**Published:** 2017-01-26

**Authors:** Carla Mucignat, Antonio Caretta

**Affiliations:** 1 Department of Molecular Medicine, University of Padova, Via Marzolo, 3, 35131 Padua, Italy; 2 INBB, National Insitute of Biostructures and Biosystems, Rome, Italy; 3 Department of Pharmacy, University of Parma, Parma, Italy

**Keywords:** Parkinson’s disease, Animal models, Olfactory bulb, Protein kinase A

## Abstract

**Background:**

Olfaction is often affected in parkinsonian patients, but dopaminergic cells in the olfactory bulb are not affected by some Parkinson-inducing drugs. We investigated whether the drug MPTP produces the olfactory deficits typical of Parkinson and affects the olfactory bulb in mice.

**Findings:**

Lesioned and control mice were tested for olfactory search, for motor and exploratory behavior. Brains and olfactory mucosa were investigated via immunohistochemistry for thyrosine hydroxylase, Olfactory Marker Protein and cyclic AMP-dependent protein kinase as an intracellular pathway involved in dopaminergic neurotransmission. MPTP induced motor impairment, but no deficit in olfactory search. Thyrosine hydroxylase did not differ in olfactory bulb, while a strong decrease was detected in substantia nigra and tegmentum of MPTP mice. Olfactory Marker Protein decreased in the olfactory bulb of MPTP mice, while a cyclic AMP-dependent protein kinase increased in the inner granular layer of MPTP mice.

**Conclusions:**

MPTP mice do not present behavioural deficits in olfactory search, yet immunoreactivity reveals modifications in the olfactory bulb, and suggests changes in intracellular signal processing, possibly linked to neuron survival after MPTP.

## Background

In human Parkinson’s disease (PD) patients, an impairment in the sense of smell and in olfactory structures is often reported [1, 2]. Neurons of the olfactory system are affected by degenerative changes, like the presence of Lewy bodies [3, 4] at an early stage, when motor deficits are not yet apparent: hence, modifications of the olfactory system can be used for early PD diagnosis [5].

Olfactory processing is linked to dopaminergic signaling, which has a prominent role in the olfactory bulb (OB) circuitry: dopamine D2 receptors in terminals of olfactory neurons and in dendrites of mitral/tufted cells modulate glutamate release, and in terminals of GABAergic/dopaminergic cells they modulate GABA and dopamine release [6, 7]. Moreover, in olfactory neurons dopamine inhibits adenylyl cyclase [8] and in OB granule cells activation of D1 receptors modulates GABA A receptors through the cAMP/protein kinase A (PKA) activation [9].

Dopamine and cAMP signalling pathway mutually interact also in brain nuclei involved in PD. PKA stimulates dopamine uptake [10], and activates tyrosine hydroxylase (TH) [11]. On striatal GABAergic neurons, D1 receptor activates PKA, that phosphorylates glutamate NMDA receptors [12]. PKA regulates dopamine physiology and modulates the activity of proteins involved in PD, including LRRK2, alpha-synuclein, tau and TH [13–16].

The OB is one of the main dopaminergic nuclei in the brain [17]. It receives axons from new receptors that continuously differentiate in the olfactory neuroepithelium, and new cells from the subventricular zone, that become periglomerular and granular cells. Dopamine is a paracrine signal for differentiation of subventricular stem cells [18], therefore a deficit in dopaminergic cells acts also on neuron turnover in the OB [19]. Moreover, cAMP regulates differentiation and survival of new neurons in the OB [20].

To better understand whether mesencephalic and OB dopaminergic neurons respond differentially to chemical insults, we investigated the changes in TH and PKA in the OB of the murine PD MPTP (1-methyl-4-phenyl,1,2,3,6-tetrahydropyridine) model.

## Methods

### Animals and treatment

Experiments were authorized according to the directive 86/609/EEC. Twelve C57BL/6j male mice 4 months old were used (Charles River, Lecco, Italy). MPTP hydrochloride (Sigma, Milan, Italy; 15 mg/kg in 0.9% NaCl, n = 6) or saline solution (0.9% NaCl, n = 6) was i.p. injected, four times every 2 h.

### Behavioral tests

Mice were weighted, evaluated for neurologic deficits and tested 5 days before and five after injections. On the day of injections, tests started 1 h after the last injection.

The open field test measures locomotion and exploration. The mouse was introduced in a cage (55 × 33 × 20 cm) for 10 min and videotaped. A software (Smart 2.5, 2B Biological Instruments, Varese, Italy) calculated distance, resting time, and number of rearings on the walls [21]. Thigmotaxis was quantified by measuring the permanence time and the time spent resting in proximity of the walls, excluding a central area (35 × 16 cm).

The pole test detects bradykinesia [22]: the mouse was placed on the top of a pole (1.5 cm diameter, 50 cm height). The time until it reached the floor was recorded (maximum 3 min).

The grip test [22] consisted in placing forepaws on the middle of a wire, 2 mm × 90 cm, 15 cm above the floor: the time to fall down or to reach the lateral platforms was recorded (maximum 3 min).

The cookie-finding test evaluates olfactory function [21]. On the second day pre-injection and after injection, mice were overnight deprived of food then put in a cage (42 × 25 × 15 cm) with a food pellet buried under the sawdust: the latency to discover it was recorded within 5 min. The test was repeated after 1 h with a pellet in a visible position to control for motivation to eat. The test was not repeated every day in order to avoid unnecessary stress due to overnight food restriction.

The tests were repeated on all mice up to day 3 post-injection, then half of the mice were sacrificed, on day 4 and 5 three mice were tested in each group. The data collected before treatment were compared to those collected after injection: data were analyzed with mixed design analysis of variance (ANOVA, factors Group: control/MPTP; Day: pre- vs. post-treatment) and post hoc Newman–Keuls, using Statistica 5 software (www.statsoft.com). The significant level was p < 0.05. Data from behavioral tests are presented as mean ± SEM.

### Immunohistochemistry

Three or 5 days after injection, mice were euthanized (Tanax 20 mg/kg, i.p.). After preincubation of paraffin-embedded sections for 1 h with 2% bovine serum albumin, nose and brain sections were incubated overnight with Olfactory Marker Protein (OMP) antibody (Wako, Neuss, Germany, 1:600); brains were also incubated with TH (Santa Cruz Biotechnology, Heidelberg, Germany, 1:100), or synaptophysin antibodies (Sigma, Milan, Italy, 1:100). Frozen sections were washed for 30 min in 2% Triton-X100, fixed for 1 min in formalin at 37 °C and incubated overnight with anti- murine PKA RIIalpha (Santa Cruz Biotechnology, Heidelberg, Germany, 1:200); adjacent sections were incubated with 100 nM 8-thioacetamido-fluorescein-cAMP (SAF-cAMP) to visualize PKA RI [23]. Secondary antibodies (Sigma, Milan, Italy; or Molecular Probes, Milan, Italy) 1:400 were incubated 1 h at 37 °C. Slides were evaluated independently with a Leica microscope (objectives: 20×, 40×, 100×) by two observers on a semiquantitative 5-step scale. Unaltered images obtained using the same conditions were mounted with Corel Draw 12 (Corel Corporation, Ottawa, Canada).

## Results

### Behavioral tests

Mice did not differ in body weight, before and after the treatment: before treatment, 25.1 ± 1.1 versus 24.9 ± 1.1 g for controls and MPTP-treated, respectively; after treatment: 24.2 ± 1.1 versus 24.4 ± 1.2 g for controls and MPTP-treated, respectively.

**Fig. 1:**
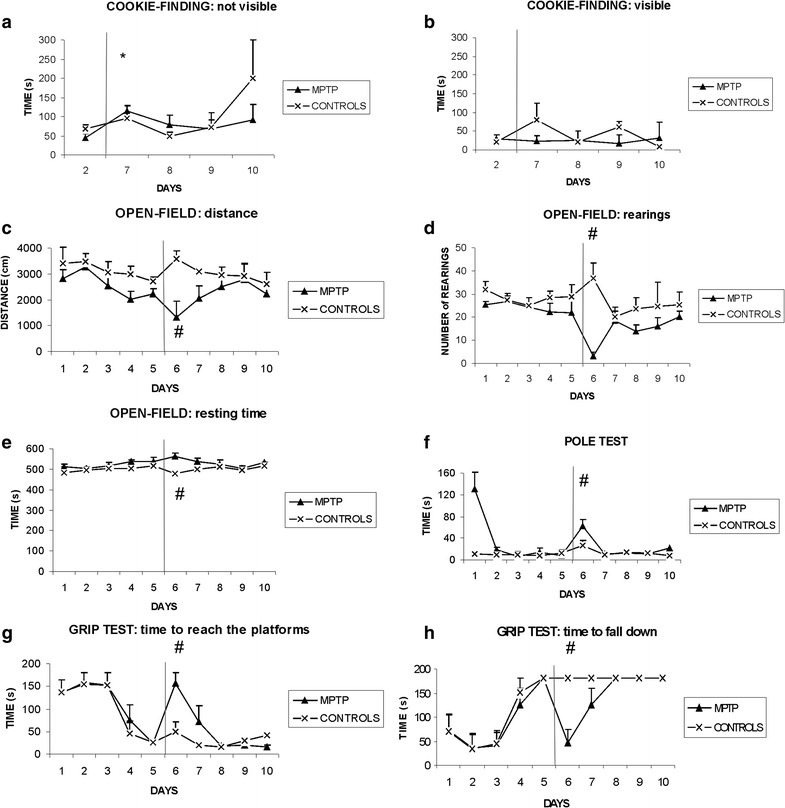
Time course of behavioral performance in different tests. Control mice (*times symbol*) and MPTP mice (*filled triangle*) mean ± SEM were compared between groups on day 5 (last day before treatment) and day 6 (after treatment). The *vertical line* indicates the time of injections, *asterisk* indicates that both groups were different from the pre-treatment, *hash symbol* indicates that MPTP mice differ from controls after treatment. **a** Cookie-finding test, with the food pellet hidden. **b** Cookie-finding test with the food pellet in a visible position. **c** Open field test, distance travelled. **d** Number of rearings on the walls during the open field test. **e** Cumulative time spent resting in the open field test. **f** Time to reach the ground in the pole test. **g** Grip test, time to reach effectively the extremity. **h** Grip test, time to fall down

All mice during the first and second repetition of the tests improved their performance, to reach a steady-state performance before the treatment (Fig. 1). After the treatment, MPTP-treated mice displayed some deficits, in comparison to controls and to their pre-treatment scores.

The Cookie-finding test did not differ between controls and MPTP mice when food was hidden. Only the factor Day was significant, F(1,10) = 6.995, p < 0.05: both groups after treatment were slower in retrieving hidden food, possibly as a consequence of the stress imposed by injections. MPTP mice did not display any obvious deficit with visible food. All mice were able to retrieve the food in both invisible and visible conditions.

A reduction in motor activity after MPTP was apparent in the open field, with a decrease in the distance travelled [Group: F(1,10) = 20.094, p < 0.005 controls: 3136.9 ± 214.8 cm, MPTP: 1776.4 ± 214.3 cm] and in the number of rearings; the significant interaction Group × Day, F(1,10) = 7.817 p < 0.05, showed that only MPTP mice performed less rearings after the treatment, 28.6 ± 5.4 and 36.8 ± 6.4 rearings for controls (p = 0.25), and 21.8 ± 5.6 versus 3.0 ± 1.8 rearings in MPTP mice (p < 0.005). The resting time differed between groups, F(1,10) = 22.834, p < 0.001, with MPTP mice resting for a longer time compared to controls, 550.0 ± 7.6 versus 497.2 ± 7.9 s.

By analysing only to the zone adjacent to the walls, the factor Day was different, F(1,10) = 71.838, p < 0.00001: both control and MPTP mice spent a longer time (including walking and resting) along the walls after the treatment, compared to the day before treatment (p < 0.005). This may be due to the stress of injections. Considering only the time spent resting in the proximity of the walls, both the factors and the interaction were significant. The interaction Group × Day, F(1,10) = 9.952 p < 0.02, showed that while controls did not vary (360.2 ± 16.3 vs. 387.8 ± 17.5 s, respectively), MPTP mice spent a longer time resting along the border after the treatment (386.6 ± 16.9 vs. 522.5 ± 29.6 s before and after the treatment respectively, p < 0.001).

In the pole test, the factor Day was significant, F(1,10) = 13.673, p < 0.005, and the interaction tended to significance, F(1,10) = 4.749, p = 0.054: only MPTP mice were slower in reaching the ground after the treatment (7.3 ± 2.7 vs. 62.1 ± 12.2 s), while controls did not vary significantly (11.8 ± 7.0 vs. 26.0 ± 9.0 s).

In the grip test, the time to reach one end was different: the significant interaction, F(1,10) = 12.597 p < 0.01, showed that only MPTP mice took longer to reach the extremity after the treatment (24.1 ± 6.7 vs. 156.6 ± 23.3 s in MPTP mice, p < 0.001, and in controls: 25.5 ± 5.7 vs. 49.8 ± 20.5 s). Only mice treated with MPTP fell down (5 out of 6 mice), as shown by the interaction Group × Day, F(1,10) = 24.217, p < 0.001.

### Immunohistochemistry

**Fig. 2:**
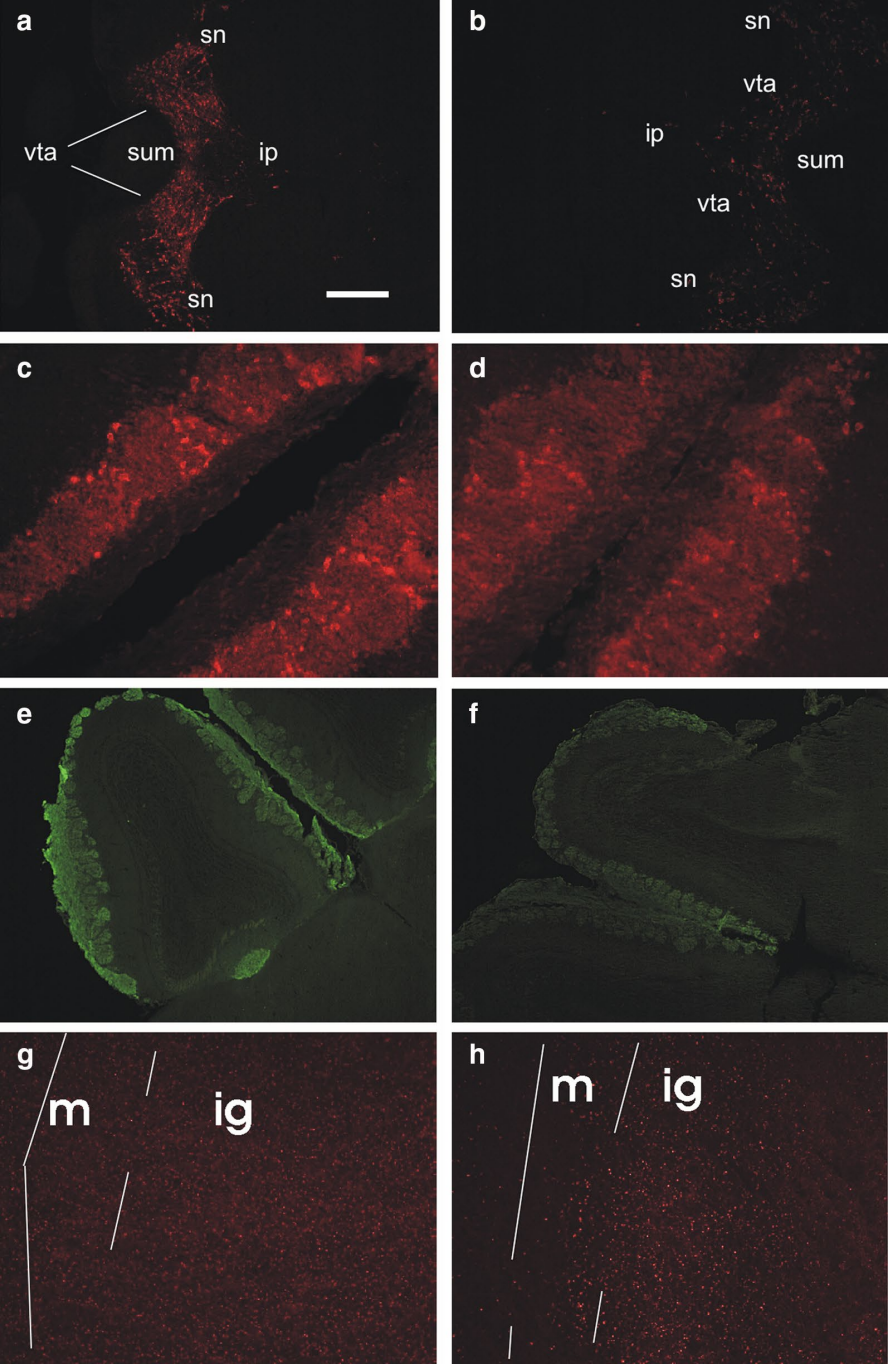
Immunohistochemistry on horizontal brain sections. *Bar* 200 μm for **a**, **b**, **e**, **f**; 50 μm for **c**, **d**, **g**, **h**. **a**–**d** TH immunolabelling. **a** Substantia nigra/ventral tegmental area appear labelled in a control mouse; caudal on the *right*. *ip* interpeduncular nucleus, *sn* substantia nigra, *sum* supramammillary nucleus, *vta* ventral tegmental area. **b** The same area is almost unlabelled in a MPTP mouse, 3 days after injections; rostral on the *right*. **c**, **d** Periglomerular cells are similarly labelled in a control mouse (**c**) and in a MPTP-treated mouse (**d**), 3 days after injections; caudal on *top right*. **e**, **f** OMP immunoreactivity in the olfactory bulb of a control (**e**) and a MPTP mouse (**f**), 3 days after injections. In the control mouse, the olfactory nerve and glomerular layer are labelled; also the accessory olfactory bulb glomerular layer is labelled. Caudal on the *lower right*. **g**, **h** RII immunoreactivity in the main olfactory bulb of a control (**g**) and a MPTP-treated mouse, 3 days after injections. Caudal on the *top*, lateral on the *left*. *m* mitral cell layer, *ig* inner granular layer

Data on immunohistochemistry are summarized in Table 1 and representative sections are shown in Fig. 2. As expected, TH was reduced in substantia nigra after MPTP (Fig. 2a, b), but did not change in the OB (Fig. 2c, d).

**Table 1:** OMP and TH immunostaining

	Control	Day 3	Day 5
OMP			
Olfactory nerve	+++	+	+
Olfactory glomeruli	+++	+	+
TH			
Olfactory bulb	++	++	++
Substantia nigra	+++	±	±

No labeling (−); faint labeling (±); moderate labeling (+); intense labeling (++); very intense labeling (+++)

In the nose of both groups, OMP labelling was prominent in olfactory neurons, olfactory fila, and vomeronasal neurons. However, the olfactory nerve and glomeruli in the main OB were consistently fainter in MPTP mice (Fig. 2e, f). This difference was not present in the accessory OB. Synaptophysin did not change in both control and MPTP mice.

No obvious modification was apparent in PKA RI, yet 3 days after MPTP PKA RII was brighter in the inner granular layer of the main OB and in the granule cells of the accessory OB, compared to controls (Fig. 2g, h, see also [23]). Five days after MPTP, this difference was less apparent, the most intense granule cells were confined immediately below the mitral cell layer, while the inner granular layer was very faint.

## Discussion

Multiple factors contribute to the onset and progress of PD, that initially targets few susceptible neuron types in motor nuclei of glossopharyngeal and vagus nerves, and in the anterior olfactory nucleus [2, 3, 24]. Many animal models are available for mimicking PD landmarks, however none can reproduce in full the human pathology. The picture is even more complicated by the presence of both motor and non-motor symptoms, whose assessment in animal models may be difficult and need careful overall interpretation (for a review, see [25]). We choose the MPTP model because we were interested in olfactory dysfunctions, which are a hallmark of early PD stages. MPTP administered acutely in mice can reproduce early stages of PD [26], however, it is not sufficient for fully exploring PD, since MPTP-injected mice recover spontaneously, which precludes the study of pharmacological interventions. Moreover, various strains of mice show different sensitivity to MPTP.

PD implies a complex imbalance of the dopaminergic system. However, not all dopaminergic neurons in the brain are equally affected by degeneration.

Here, in MPTP mice olfactory search behavioural performance is normal, yet some modifications can be detected via immunoreactivity in the OB.

MPTP in mice induces both behavioral and neurochemical changes that mimic human PD: altering free radicals quenching and reducing TH and dopamine transporter in the substantia nigra and striatum [27] result in motor impairments, bradykinesia and catalepsy [22]. However, MPTP does not worsen olfaction in both humans and mice [28, 29]. In our experiment, mice were impaired in motor performance, as shown by short travelled distance and less rearings on the walls—an increase in the rearings is used as an index of anxiety. However, MPTP mice performed similarly to controls in olfactory retrieving, which also included motor performance. This may be due to differential involvement of the motivational system, which is conceivably more activated in discovering and reaching food items. PD patients are impaired in the motor but also in cognitive/integrative levels of motor control [30], and often show apathy and indecisiveness [31]: similarly, our mice move under a sufficiently strong drive. However, their good performance in the cookie-finding test does not imply a normal olfactory function. This test involves exposure to above-threshold stimuli: it is possible that MPTP mice show subtler olfactory deficits. Noteworthy in a genetic PD model, mice were able to detect and habituate to odors, but showed deficits in more stringent olfactory tests [32].

MPTP modifies the expression of several proteins: in the striatum it reduces dopamine, TH, dopamine transporter, vesicle monoamine transporter and alpha-synuclein, while monoaminooxidase A and B and catechol-O-methyl-transferase remain unchanged [33]. Reduced TH levels were apparent in our MPTP mice in the brainstem dopaminergic nuclei.

The dopaminergic pathway is linked to cAMP intracellular signalling in both brainstem and olfactory system: TH is induced by the cAMP-mediated signalling pathway [34]. In PD substantia nigra, both D1 receptors and DARPP32 appear downregulated [35]. Dopaminergic neurons are protected against MPTP toxicity after the inhibition of monoaminooxydase-B, which acts via PKA [36]. Moreover, substantia nigra is protected from MPTP by phosphodiesterase inhibitors, that enhance cAMP and subsequently PKA [37]. Here, the transient increase in OB PKA after MPTP may be linked to a protective response in these neurons, opening a new challenge for neuroprotection in other brain areas.

PKA participates also in reaction to MPTP, so that in lesioned mice activation of PKA with forskolin induces an exaggerated increase in TH [38]. Moreover, glutamatergic corticostriatal pathway is overactive after nigrostriatal denervation, and subsequently striatal PKA-dependent NMDA phosphorylation increases [39].

Striatal deafferentation increases neurogenesis in the olfactory bulb, mostly for new dopaminergic cells [40, 41]. In our MPTP mice, the PKA transient increase in OB inner granular layer, which hosts also the developing new neurons coming from the subventricular zone, suggests an upregulation of the cAMP-mediated signalling in response to MPTP, which should be studied in greater detail.

The differential effects of MPTP in OB neurons were related to the lack of the dopamine transporter, which uptakes the toxic MPTP metabolite, making OB neurons MPTP-resistant [42]. While further studies, including other animal PD models like 6-OHDA, are needed, the present data confirm that TH immunoreactivity is not affected in the OB after MPTP injection. However, we challenge the idea that the olfactory system is not affected by MPTP at all, since a decrease in OMP and a transient increase in PKA RII were consistently observed, suggesting a specific response to MPTP in OB neurons, that is not apparent in the nigra, and could be exploited for therapeutic purposes.

## Abbreviations

ANOVA: analysis of variance
GABA: gamma-aminobutirric acid
MPTP: 1-methyl-4-phenyl-1,2,3,6-tetrahydropyridine
OB: olfactory bulb
OMP: Olfactory Marker Protein
PBS: phosphate buffered saline
PD: Parkinson’s disease
PKA: cAMP-dependent protein kinase
R: regulatory subunit of PKA
SAF-cAMP: 8-thioacetamido-fluorescein-cAMP
TH: tyrosine hydroxylase
